# Extracts from *Frangula alnus* Mill. and Their Effects on Environmental and Probiotic Bacteria

**DOI:** 10.3390/plants11202719

**Published:** 2022-10-14

**Authors:** Agata Kledecka, Przemysław Siejak, Anubhav Pratap-Singh, Przemysław Łukasz Kowalczewski, Farahnaz Fathordoobady, Maciej Jarzębski, Wojciech Smułek

**Affiliations:** 1 Institute of Chemical Technology and Engineering, Poznan University of Technology, 4 Berdychowo Str., 60-965 Poznań, Poland; 2 Department of Physics and Biophysics, Poznań University of Life Sciences, 38/42 Wojska Polskiego Str., 60-637 Poznań, Poland; 3 Food Nutrition and Health Program, The University of British Columbia, 2205 East Mall, Vancouver, BC V6T 1Z4, Canada; 4 Department of Food Technology of Plant Origin, Poznań University of Life Sciences, 31 Wojska Polskiego Str., 60-637 Poznań, Poland

**Keywords:** anthraquinones, frangulin, buckthorn, biomembrane, permeability

## Abstract

The bark of *Frangula alnus* Mill (FAM), the so-called alder buckthorn, has been widely investigated for its medicinal properties, especially its laxative effects and the bioactive properties of the plant material extract. Still, there is no wider study devoted to its antibacterial properties. This is important in the context of its impact on probiotic gut bacteria. The aim of the research was to recognize the effect of FAM extract on bacterial cells, and to determine how the bioactive properties and composition of the extract are influenced by the type of solvent used for the extraction. To find the most suitable conditions for the FAM extraction, we used four solvent solutions with different polarities, including water, methanol, ethanol, and isopropanol. We assessed the quality and composition of the extracts with spectral analysis, using spectrophotometric (FTIR, UV-Vis) and chromatographic methods (GC-MS). Finally, we analyzed the extractant impact of the extracts on the selected bacterial cells. The results showed that the chemical diversity of the extracts increased with the increase in solvent polarity, in which the abundance of frangulin, the main bioactive compound in buckthorn bark, was confirmed. *Pseudomonas fluorescens* ATCC 17400 was particularly sensitive to the action of extracts, whereas other strains of the *Pseudomonas* genus showed practically no adverse effects. Ethanolic extracts had the strongest effect on most of the selected bacteria strains. We found that the probiotic *Lactobacillus* strain, which represents intestinal microflora, has no direct effect on probiotic microorganisms. The research shown FAM extracts can be safe for probiotic bacteria present in human gut microflora. Moreover, the study indicated that contact with the extracts may reduce the total permeability of the bacterial membranes. This opens up the possibility of using FAM extracts as a factor regulating transport into cells, which may be used to support the action of other bioactive substances.

## 1. Introduction

There is a growing demand for new types of drugs, dietary supplements, and other kinds of preparations containing biological active compounds from natural raw materials, especially plant species that are easily available and cost-effective [1,2,3,4]. Buckthorn bark (*Frangula alnus* Mill, also known as *Rhamnus frangula* Linn.) is a raw herbal material that has been used in folk medicine for centuries [5,6]. Its abundance was discovered (among other medicinal plants) in the ruins of the ancient city of Dascyleum (Daskyleion) in the province of Baikesir in northwestern Turkey [7]. For at least several centuries until today, it has been a popular herbal resource in Europe, including British Islands [8], the Carpathian region [9], and the Balkan Peninsula [10,11]. Its medicinal properties and safety of use have been approved by the European Medicines Agency (EMEA) and the Committee on Herbal Medicinal Products (HMPC) for internal use [6].

Glossy buckthorn (*F. alnus* Mill) is a shrub of the Rhamnaceae family and is commonly used as an herbal raw material. It belongs to nanophanerophytes, a subgroup of cold-hardy shrubs and perennials less than two meters high. The fruit of the shrub is considered poisonous and unpalatable to humans, while it is food for many species of birds. The shrub does not contain thorns on its twigs, so it can be distinguished from a similar species of the shrub [8,12,13]. In the past, the bark of the bush was used for the production of gunpowder and charcoal. Fruits and, in some cases, buckthorn bark have been used to dye various types of materials, for example, wool: yellow-green, purple, or yellow-brown [14].

Anthranoid compounds, including frangulin, have significant pharmacological properties. They show antioxidant, antibacterial, fungicidal, antiviral, and anticancer properties, and are used in medicine as laxatives (mild agents laxantia or strong agents purgation). One of the anthracompounds, hypericin, is also recognized as a substance that helps to cease the symptoms of depression [5,15,16].

The potency of laxatives depends on the structure of the anthranoid molecule. Compounds containing a larger number of sugar molecules have a stronger effect. The most potent laxatives are anthrone and dianthrone compounds. Anthranoid substances are very rarely administered in the form of isolated compounds or galenic preparations. Most often, herbal infusions are used or served in the form of tableted extracts. Anthracompounds are absorbed from the small intestine into the blood, then quickly passed through the mucosa to the large intestine, where they are hydrolyzed and reduced to active forms of anthracompounds via reactions of enzymes and bacteria. The walls of the large intestine (Auerbach’s cells) become irritated, which intensifies peristaltic movements and the displacement of residual food content. At the same time, the secretion of water into the lumen of the large intestine increases. The resorption of water from the large intestine is inhibited. As a result, the digestive contents become more fluid. The effect of the anthranoid compounds is observed 8–12 h after consumption, as they only work after reaching the large intestine. Depending on the plant extract used, the onset of the action of the laxative can be observed differently, e.g., for aloe vera (2–6 h), and differently for *Rhamnus catharica* (6–8 h). The major differences depend on the contents of the anthranoid compounds present in a given plant species. Large anthracompound molecules and those that are not decomposed in the acidic environment of the stomach pass quickly to the large intestine. They do not decompose in the upper gastrointestinal tract. On the other hand, anthranoids absorbed in the stomach and free aglycones reach the large intestine only after detoxification in the liver and secretion with bile into the duodenum [17]. Despite the variety of studies on *F. alnus* Mill bioactivity, there is a lack of information on how the extracts may affect the probiotic and other bacteria strains.

The used solvent and extrahent for the plant extract preparation might play a crucial role for their final properties, as well as amount of desirable compounds/active agents [18,19]. For example, Kukula-Koch et al. showed that for horse chest nuts extract, a high antioxidant potential was registered for methanolic extracts [20].

In this study, we focused on the antibacterial activity of the *F. alnus* Mill (FAM) extracts and their threat to probiotic gut bacteria, and the impact of the solvent used as an extractant on extract bioactivity. We used four liquid extractants with different polarities: water, methanol, ethanol, and isopropanol. The qualitative differences of plant extracts depending on the solvent used were determined using Fourier-transform infrared spectroscopy (FT-IR) and UV-VIS spectrophotometry. Additionally, chromatographic methods, i.e., gas chromatography coupled with mass spectrometry (GC-MS) were also used to analyze the samples. The second part of our examinations was to verify the impact of the extractant on the bioactivity of several bacterial strains, including the study of the metabolic activity of cells and membrane permeability tests. The results presented can be used for the next steps of the studies, a detailed study of antimicrobial activity of compounds identified in FAM extract.

## 2. Materials and Methods

### 2.1. Chemicals

All solvents, including acetone and alcohols, were provided from Avantor, Poland. Other chemicals were of analytical or chromatography grade, and were purchased from Sigma-Aldrich, Poland. Microbiological nutrients were purchased from BTL Sp. z o.o., Poland. Dried and cut *F. alnus* Mill bark was purchased as herbal material from Flos, Poland.

### 2.2. Extraction Procedure

Plant material was extracted with water, methanol, ethanol, or isopropanol as follows: 5 g of cut *F. alnus* Mill bark was put in a cellulose tube and placed in a Soxhlet extractor filled with 200 mL of solvent. The apparatus was heated on a laboratory electric heating mantle for 7 h. Next, the samples were filtered and the solvent was evaporated using a rotary evaporator R-210 (Büchi, Flawil, Switzerland). Purified extract was freeze-dried for 24 h at −55 °C under a pressure of 0.37 mbar (Freeze Dryer Alpha 1-2 LD Plus, Christ, Osterode am Harz, Germany). Crude extracts were stored at 4 °C.

### 2.3. Extracts Analysis

The differences between chemical compositions were analyzed using spectroscopic methods. To obtain the UV-VIS spectra (200–900 nm) 0.8 g L^−1^ solutions of the extract were prepared and analyzed using SkyScanner (Thermo Scientific, Japan). The infrared spectra (400–4000 cm^−1^) were prepared in KBr discs using Vertex 70 (Bruker, Bremen, Germany) FTIR spectrometer.

The content (%) of glucofrangulin (A or B) in plant extract solutions after overflow extraction processes were reported based on the intensity of the maximum absorption at the maximum wavelength of λ_max_ (Table 1). The calculations were based on $\varepsilon =10^{4.52}$ dm^3^·mol^−1^·cm^−1^ [21], which refers to λ_max2_. Moreover, GC-MS analysis was conducted according to a procedure as follows. The research used the Pegasus 4D GCxGC-TOFMS system by LECO, and the MultiPurpose Sampler by GERSTEL. The studies were conducted in a one-dimensional model using a universal SGE BPX5 (5% phenyl, 95% PDMS) column. Spectra obtained via electron ionization (EI-source) were analyzed using the NIST mass spectral library. The GC-MS analysis took into account the background of the spectrum constituting the masses of compounds from the liquid phase of the column and the presumed air compounds [22]. For GC-TOFMS analysis, plant samples were prepared by adding BSTFA silylation reagent or acetic anhydride after dissolving the plant extracts in *n*-hexane [23]. The probability of the proposed compounds had to be at least 60%.

### 2.4. Biological Activity of Extracts

Two environmental strains, *Pseudomonas plecoglossicida* (GenBank No. KY561350) *Pseudomonas* sp. MChB (GenBank No. KU563540), and three standard strains, *Pseudomonas fluorescens* 17400 ATCC and *Lactobacillus rhamnosus* 492 PCM, were applied. The *Pseudomonas* strains were cultivated in nutrient broth, and for the *Lactobacillus* strain, MRS broth was used. The strains were incubated 24 h at 30 °C, then centrifuged (5 min, 4500 rcf), and the cells were re-suspended in phosphate buffer at pH 7.2.

For the toxicity of the extracts against the tested strain, the MTT (3-(4,5-dimethylthiazol-2-yl)-2,5-diphenyltetrazolium bromide) assay was implemented according to Smułek et al. [24]. The changes in bacteria total cell membrane permeability were investigated with the crystal violet method described by Pacholak et al. [25]. Moreover, the inner membrane permeability was analyzed using the ONPG (o-nitrophenyl-β-galactoside) test according to Smułek et al. [26].

### 2.5. Statistical Analysis

The statistical analysis was performed with MS Excel 2013 (Microsoft Corp., Redmond, WA, USA). All experiments in this study were performed in triplicate, and the mean values and standard deviations were calculated. Statistical analysis of the correlation of the results was performed using one-way analysis of variance (ANOVA), with *p* < 0.05.

## 3. Results

### 3.1. The Efficiency of the Extraction Process from the Bark of F. alnus Mill

The efficiency of the overflow extraction process from buckthorn bark is presented in Table 1. Four different extrahents were used in the overflow extraction process to test the difference in extraction efficiency. Depending on the solvent used, four extracts were obtained with a yield not exceeding 20%. It was found that higher values of extraction efficiency were obtained for more polar solvents. The highest process efficiency of 19.78% was obtained with ethanol, followed by methanol and isopropanol efficiencies of 19.23% and 9.61%, respectively). It seems that anthranoid compounds are more soluble in alcoholic solutions with higher polarity. In the case of using distilled water, the yield was 12.22%, which also confirms the influence of the polarity of the solvent on the extracted product, and indicates a moderate solubility of the anthracompounds in distilled water.

### 3.2. Extracts Composition

The registered UV-VIS spectrum in the range of 200–900 nm wavelength with no significant observed signals above 500 nm (results non shown). For the evaluation of the glucofrangulin content, we based this on the method presented in [21]. The absorption peak maxima Amax_1_ and Amax_2_ correspond to the glucofrangulin content and ranged from 0.12 to 0.34%. The highest values were registered for the ethanol extract (0.20–0.34%), and the lowest for the isopropanol extract (0.12–0.31%).

**Figure 1:**
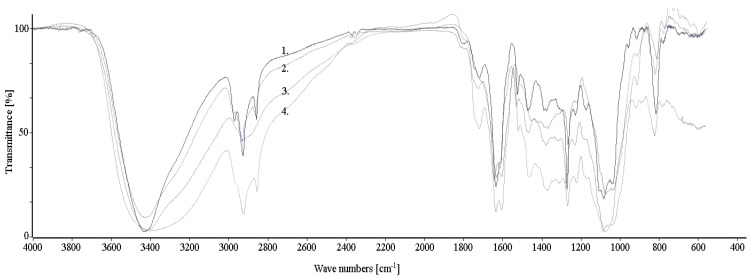
FT-IR spectra for *F. alnus* Mill extracts extracted with: 1. isopropanol, 2. ethanol, 3. distilled water, 4. methanol.

The impacts of different solvents for the FTIR spectra are presented in Figure 1. For the 3450 cm^−1^ wavenumber, there are strong and broad signals originating from the hydroxyl groups, indicating intermolecular bonds. After extraction with methanol, a distinguished, broadened signal shape in this wave range was observed. In the range from 2950 to 2800 cm^−1^, a triple signal was observed (the best split signal for the spectrum after extraction with isopropanol, the weakest split signal for the spectrum after extraction with distilled water), which indicates the presence of C-H stretching bonds, and may suggest a methylene group and a small share of the carbonyl group (C=O bonds). The band of the carbonyl group has a much wider range, from 3200 to 2400 cm^−1^. Confirmation of this group was also found with a strong signal in the wavenumber range of 1620–1600 cm^−1^ and a smaller one at the 1280 cm^−1^ wavenumber, indicating the presence of aromatic conjugated ketone groups. The confirmation of C-H bonds was also found at 1480, 1400 cm^−1^, and the range of 1200–800 cm^−1^. A double signal with a deforming structure at the level of 1620–1600 cm^−1^ may also indicate the presence of a hydroxyl group. The wavenumbers of 1649 and 1678 cm^−1^ may indicate tensile vibrations, and thus, the presence of six-heterocyclic ring structures. The last observed signals in the range of 900 to 675 cm^−1^ may show C-H bending vibrations outside the molecular plane, indicating the presence of polycyclic aromatic structures.

The identification of saturated hydrocarbons, methylene groups, and hydroxyl and carbonyl groups, and the appearance of conjugated six-heterocyclic ring structures becomes possible for four plant extracts. The spectra are slightly different from each other and can be superimposed in most fragments. The difference is in the strength of the signals observed at a given spectrum. In contrast to the well-separated isopropanol spectrum, the signals were weakly separated in an ethanol and water-based spectrum. The spectrum for the methanol-based extract is the most distinguished of all the analyzed extracts. The stretching of the signals, especially for vibrations in the range of 3700–3000 cm^−1^, may be caused by the overlapping of hydrogen bonds. The more concentrated the analyzed extract, the more the bands assigned to hydroxyl groups stretching appears and a shift towards lower wavenumber values can be observed. Sharp bands of the hydroxyl group in this range can be mainly observed for the gas phase, nonpolar solvents, and more dilute solutions. Due to the higher polarity of methanol, the band shape becomes blurred and a shift towards longer wavenumbers can be observed. For the aqueous extract, there is also a strong blurring in this range and a smaller isolation of the signal, caused by the relatively poor solubility of anthranoid compounds in water. The less polar the alcohol solution is, the better the sharpening of signals and the better the separation of the analyzed spectrum. The best resolution was obtained for the isopropanol extract.

Buckthorn bark plant extracts may also contain derivatives of the tetracyclic cholestane and cholate compounds, which may presumably be derived from phytosterols present in *F. alnus* Mill. The identified tetracyclic compounds are listed in Table S1. Compounds that were duplicated in extracts are presented in Table S2. This means that the presence of these compounds in plant extracts of *F. alnus* Mill can be assumed. Table S3 also shows the presence of single chemical compounds for the ethanol extract and the BSTFA reagent, as well as the methanol extract and the BSTFA reagent, i.e., for the chemical compounds that are unique in other plant extracts.

The analysis of chromatograms of plant extracts from buckthorn allowed us to determine the potential content of anthraquinone compounds, steroids, phytosterol derivatives, and chemical compounds with antibacterial, antifungal, or bacterial cell nourishment properties, as well as chemical compounds that are used as drugs, antibiotics, or even toxic compounds that might be used for the synthesis of some pharmaceutics.

### 3.3. Impact of F. alnus Extracts on Bacteria

#### 3.3.1. Cell Viability

**Figure 2:**
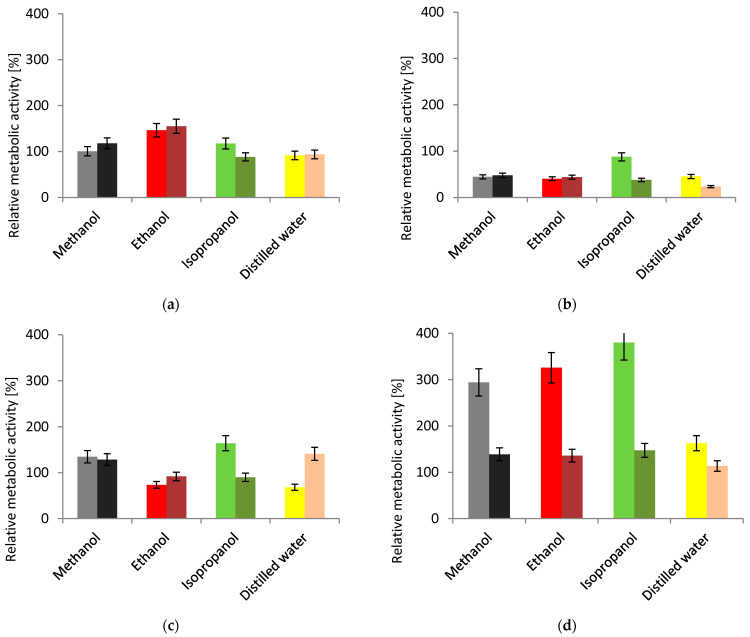
Cell viability assay; each pair of columns represents *F. alnus* Mill. extract concentrations: left, 0.5 g dm^−3^; right, 0.1 g·dm^−3^; (**a**) *Pseudomonas putida*; (**b**) *Pseudomonas fluorescens* (ATCC 17400); (**c**) *Pseudomonas fluorescens* (environmental); (**d**) *Lactobacillus rhamnosus*.

The MTT assay was performed to test the viability of cells from plant extracts of *F. alnus* Mill. In all cases (Figure 2), there was an increase in bacterial cells, regardless of the concentration of the plant extract used. The value of the relative metabolic activity increased as the number of living cells increased. Values above 100% indicated a very high degree of activity of the microorganisms compared to the control culture without the addition of *Frangula alnus* Mill extracts.

In the case of the pure strain of *Pseudomonas putida*, the calculated metabolic activity expressed as a percentage was 9%, while for samples containing nutrients and plant extracts, the activity reached >88% when isopropanol was used for extraction. With the same concentration (0.1 g·dm^−3^), the highest metabolic activity was observed for the samples after extraction with ethanol (155%).

For the *Pseudomonas fluorescens* strain (ATCC 17400), a proliferation of bacterial cells was observed compared to the strain control without the addition of plant extract. The metabolic activity of the control strain for *P. fluorescens* test was 15%. The highest growth of microorganisms (89%) was observed for the sample containing 0.5 g·dm^−3^ of plant extract obtained with isopropanol.

The metabolic activity for the control of the environmental *P. fluorescens* strain was found to be 7%. After adding *F. alnus* Mill extracts, the highest growth of microorganisms was observed compared to other microorganisms. With the same concentration (0.5 g·dm^−3^), the lowest and highest values for the metabolic activity of the samples prepared with MTT compound were found for the sample containing plant extract extracted with distilled water (68%), and the sample with plant extract obtained with isopropanol (164%), respectively.

A proliferation of bacterial cells was observed for the *Lactobacillus rhamnosus* strain compared to the strain control without the addition of plant extract. Metabolomic activity values for the tested samples containing plant extracts started from 113% for an aqueous extract with 0.1 g·dm^−3^ concentration, while the activity of the control sample for *L. rhamnosus* was found to be 12%. The highest growth of microorganisms, (380%) was observed for the sample containing plant extract obtained with isopropanol and added at a concentration of 0.5 g·dm^−3^.

The results showed the varying metabolic activity of microorganism cells and their ability to multiply in the presence of different extracts. The variety of their composition and contents of nutrients and secondary metabolites were responsible for different responses of bacterial cells to contact with them. It can be concluded that plant extracts from *F. alnus* Mill support the growth of bacterial cells of selected strains.

#### 3.3.2. Cell Membrane Permeability

The determination of changes in the permeability of bacterial cell membranes under the influence of plant extracts obtained from *F. alnus* Mill was carried out with the use of methyl violet 10B (crystal violet).

**Figure 3:**
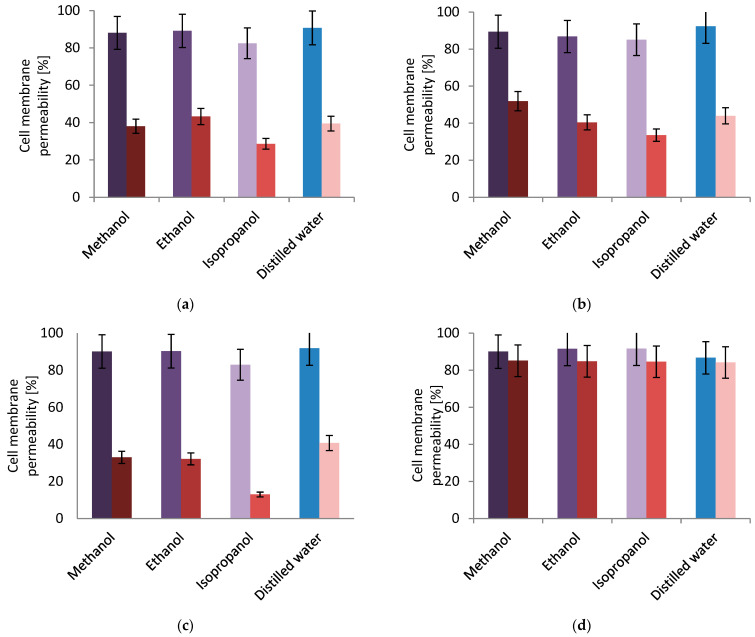
Total membrane permeability of bacterial strains; each pair of columns represents *F. alnus* Mill. extract concentrations: left, 0.5 g·dm^−3^; right, 0.1 g·dm^−3^; (**a**) *Pseudomonas putida*; (**b**) *Pseudomonas fluorescens* (ATCC 17400); (**c**) *Pseudomonas fluorescens* (environmental); (**d**) *Lactobacillus rhamnosus*.

Based on Figure 3, it was determined that the degree of dye penetration into the interior of microorganisms increased in each case with the increase in the concentration of plant extracts used. Regardless of the strain used in the tests, an increased incorporation of crystal violet into the cell can be observed in the presence of plant extracts. With *Pseudomonas putida* strain, the highest triphenylmethane dye penetration of 90% was obtained for the water extract with a concentration of 0.5 g·dm^−3^. Similarly, *Pseudomonas fluorescens* strains with 0.5 g·dm^−3^ aqueous extracts showed 92% penetration for the reference strain and 92% for the environmental strain. Different results were recorded for *Lactobacillus rhamnosus*, where the highest penetration value of crystal violet (92%) was obtained for the isopropanol extract with 0.5 g·dm^−3^. Regardless of the concentration of plant extract and the selected bacterial strain, the values of aniline dye permeation through the samples obtained after extraction with isopropanol were the lowest in each test, except for *Lactobacillus rhamnosus*, where the value was in the range of 85–92%. The lowest value of dye penetration was obtained for the environmental *Pseudomonas fluorescens* strain, with a plant extract concentration of 0.1 g·dm^−3^, and this value was 13%. Poor crystal violet penetration may indicate a higher resistance of the cells of the environmental strain which are more adapted to the varying environment. A significant difference between the degree of crystal violet penetration, depending on the concentration and the type of tested strain of the plant extract used, may indicate the resistance of bacterial cells to a certain amount of active substances present in *F. alnus* Mill. For all *Pseudomonas* strains, only when a higher concentration of the extract was used, did the permeability of cell membranes increase to over 82%. On the other hand, the results of the dye permeability for the extract concentration of 0.1 g·dm^−3^ did not exceed 52% for these strains. These results are different from what were obtained for *Lactobacillus rhamnosus*, which was a higher degree of crystal violet penetration, even with a lower concentration of plant extract (0.1 g·dm^−3^). This means that *Lactobacillus rhamnosus* is not adapted to environmental changes and is not sufficiently resistant to the penetration of crystal violet.

**Figure 4:**
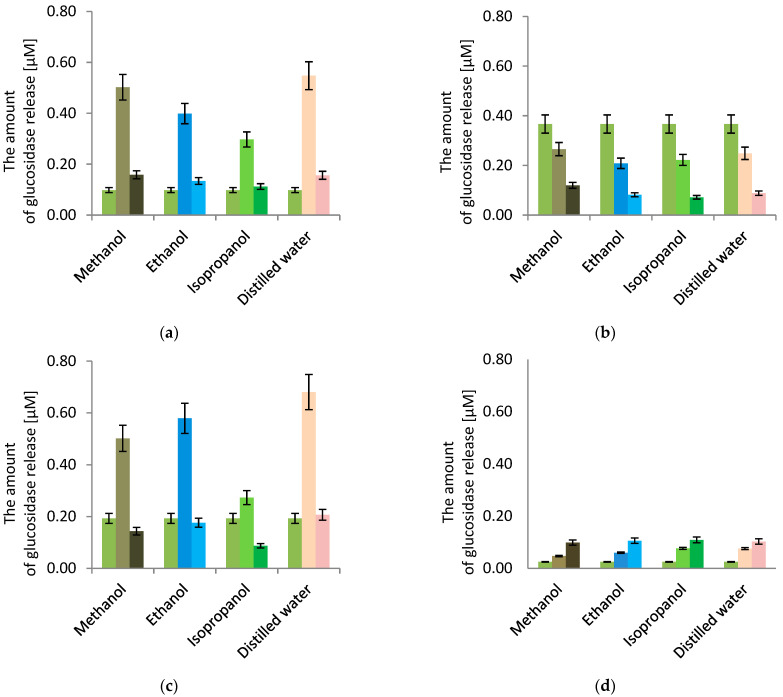
Inner membrane permeability (ONPG assay); each pair of columns represents *F. alnus* Mill. extract concentrations: left, control (0 g·dm^−3^; in center, 0.5 g·dm^−3^; right, 0.1 g·dm^−3^. (**a**) *Pseudomonas putida*; (**b**) *Pseudomonas fluorescens* (ATCC 17400); (**c**) *Pseudomonas fluorescens* (environmental); (**d**) *Lactobacillus rhamnosus*.

Figure 4 shows the results for the 2-nitrophenyl ß-D-galactopyranoside test. For each bacterial strain, the amount of chromogenic substrate determined was dependent on the concentration of the plant extract. A more intense color was observed when the concentration of plant extract from *F. alnus* Mill was 0.5 g·dm^−3^ for the *P. putida* strains and the environmental *P. fluorescens* strain. Conversely, with *L. rhamnosus*, a more intense color was observed at a lower concentration of 0.1 g·dm^−3^. Lower values were obtained for both concentrations for the reference strain *P. fluorescens* and the environmental strain *P. s fluorescens* with concentrations of 0.1 g·dm^−3^ except for the aqueous extract. Testing with *P. putida* and *L. rhamnosus* strains showed that the permeability of the inner membrane increased for all plant extracts. For the *Pseudomonas putida* strain, higher values of the dye were observed with using higher concentrations (0.5 g·dm^−3^) of plant extract, while the amount of hydrolyzed ONPG was slightly visible at a lower concentration (0.1 g·dm^−3^). This result was opposed to what was found for *L.s rhamnosus* strain, where the lower concentration led to a higher amount of chromogenic substrate. *P. putida* with water extract at a concentration of 0.5 g·dm^−3^ resulted in the greatest damage to the microbial cells, where the concentration rate of penetrating *o*-nitrophenol was 0.55 µM·min^−1^, while the isopropanol extract with a concentration of 0.1 g·dm^−3^ caused little damage to the cell membrane of *P. putida*, and the kinetics result of *o*-nitrophenol was 0.11 µM·min^−1^ compared to the control without extract, which was 0.10 µM·min^−1^.

For the reference *P. fluorescens* strain, no increase in the optical density and no color changes were observed for the samples. The concentration of *o*-nitrophenol in the control *P.s fluorescens* reference strain when the sample contained no plant extract was 0.37 µM·min^−1^. All plant extracts inhibited the formation of *o*-nitrophenol. The amount of *o*-nitrophenol colored compound did not exceed 0.27 µM·min^−1^ for samples containing the *P. fluorescens* reference strain and any extract from *F. alnus* Mill. In the presence of plant extracts, bacterial cells protect themselves against cell wall damage by reducing the permeability of the cell membrane.

A different situation for dye formation was observed with the environmental *P. fluorescens* strain. At a concentration of 0.5 g·dm^−3^, each plant extract caused an increase in the optical density of the system and an increase in the number of bacteria with damaged cell membranes. The highest amount of *o*-nitrophenol formed was observed for the sample containing the aqueous extract, and it was 0.68 µM·min^−1^. At the same time, it was the highest degree of permeability compared to all tests performed with ONPG. Using concentration of 0.1 g·dm^−3^, the amount of determined *o*-nitrophenol differed, depending on the plant extract used. Only the water extract caused a slight increase in the number of damaged internal membranes of bacteria, while methanol, ethanol, and isopropanol extracts did not damage the cell wall membranes of the environmental *P.s fluorescens* strain. The control concentration for this microorganism was 0.19 µM·min^−1^; therefore, any lower value implied a blockage of the cell membranes from the reaction of 2-nitrophenyl ß-D-galactopyranoside with β-galactosidase of the selected microorganisms.

As mentioned above, the *L. rhamnosus* strain acted exactly opposite to *P. putida* in the tested samples. The lower concentration of plant extract led to a higher response to the *o*-nitrophenol. In comparison with the control, an increase in the optical density and color changes of the samples were observed. The concentration of *o*-nitrophenol was 0.025 µM·min^−1^ when the control sample without plant extract was tested. All samples containing plant extracts exceeded the value of this control. The greatest value was obtained for an isopropanol extract with 0.1 g·dm^−3^ concentration equal to 0.11 µM·min^−1^, and the lowest value was obtained for methanol extract with 0.5 g·dm^−3^ concentration, and it was 0.05µM·min^−1^. Bacterial cells do not protect themselves against cell wall damage by reducing the permeability of the cell membrane in the presence of plant extracts, as shown in the environmental *P.s fluorescens* strain.

## 4. Discussion

Properties of biologically active compounds from *F. alnus* Mill, purchased as certified herbal materials for internal use, were determined by the tests described in this paper. The appropriate selection of extractants for overflow extraction (Soxhlet apparatus) had a significant impact on further work with the obtained extracts. The best solubility of anthranoid compounds in polar and non-toxic solvents, mainly alcohols, contributed to extracts being obtained with the highest content of anthranoid compounds due to their possible hydrophilicity. Soxhlet extraction was desirable in this case, because other methods such as sonication or maceration require either much higher extraction temperatures or an extraction time extended to several days, as described by Sasidharan et al. [27]. Moreover, extraction with a Soxhlet apparatus may protect thermolabile compounds, which was mentioned by Ong [28]. On the other hand, when comparing alternative extraction methods for the recovery of bioactive compounds from different plant materials, also cited in the publication by Nyiredy [29], one can consider extraction-assisted methods, including Microwave-Assisted Extraction (MAE) and Ultrasonic Extraction (USE). Both methods use smaller amounts of solvent and require less time to obtain the desired extract. Accelerated Solvent Extraction (ASE) is also an alternate method of extraction; however, it generates a higher temperature during the entire process and cannot be considered as a cost-effective method.

Rosenthal et al. [30] determined the concentration of plant extracts and the contents of glucofrangulin A and B using UV-VIS spectra. However, the presence of pure frangulin A and B compounds does not follow from the spectrum. In their work, the authors determined the frangulin A and B UV-Vis spectra maxima as 419.4 and 435 nm, respectively. Glucofrangulin content in plant extracts ranging from 0.12 to 0.34% is an acceptable result, considering the complexity of the whole plant extract and the variety of compounds that can be detected by GC-TOFMS. The relatively poor solubility of anthranoid compounds in water, and the polarity of the solvent itself, resulted in inaccurate signal separation in FT-IR analysis. FT-IR separation for methanolic extract with a relatively good solubility of anthranoid was not also accurate as the more non-polar solvent, causing greater sharp signals for hydroxyl groups in infrared analysis. Sharp bands of the hydroxyl group can be also observed for diluted solutions. Therefore, for more polar compounds, an attempt could be made to dilute plant samples in the case of the need of usage selected extractants for the process of extracting bioactive compounds from a given plant [1,31,32]. A good example is the separation of an isopropanol-based extract [33,34], which is less polar than methanol but still belongs to compounds with a high dipole momentum.

Chromatographic methods have not been used to investigate F. alnus Mill extract to a wide extent, so far. One of these examples is the TLC method, which was described by Stahl and Schield [35]. Comprehensive gas chromatography allows for the precise separation of co-eluting signals in three dimensions. A gas chromatography system coupled with TOFMS detector and orthogonal chromatography columns can provide the detection of possible compounds present in plant extract when there are no standards for them. In our microbiological tests, an attempt was made to find a uniform answer to the question of whether plant extracts from *F. alnus* Mill, together with the selected reagent, affected the growth of all the types of microorganisms tested. The mechanism of action towards microorganisms’ cells, by manipulating the concentration and introducing the strain into a given plant extract (ethanol-, methanol-, isopropanol-, and distilled water-based) was determined. For this purpose, the metabolic activity of bacterial cells, the permeability of the cell membrane, and the hydrolysis of the ONPG compound were studied. The results showed the complexity of the bacteria response to a given environment. Particular attention was paid to the use of similar strains of *P. fluorescens* for reference and for natural environments. In the conducted analyses, the variability of the environmental strain could be distinguished. In the MTT assay, it showed better metabolic activity than other *Pseudomonas* strains and allowed for the multiplication of subsequent bacterial cells. It should be emphasized that for *L. rhamnosus* strains, the greatest proliferation of cells occurred compared to all other strains and their plant extracts. The results exceeded 380%, indicating about cell multiplication 32-fold. However, the assay with crystal violet showed a very strong resistance by the *P. fluorescens* strain, especially against isopropanol-based extract with a lower concentration, which proved its resistance to a changing environment. However, the reference strain was less resistant to the triphenylmethane dye. The *L. rhamnosus* strain acted completely differently, with no resistance against the penetration of crystal violet dye in any concentration of the samples used. The results obtained for colored *o*-nitrophenol content in both *Pseudomonas fluorescens* strains were significantly different. The reference strain did not allow for the hydrolysis of the ONPG compound with the β-galactosidase present in the cell, compared to the control samples. The environmental strain was considered to be less resistant, as the greatest increase in the number of damaged bacterial membranes was determined, especially for the water-based extract with a concentration of 0.5 g·dm^−3^.

The application of the plant extract varies depending on the research cell selected. If there is a need to test the effect of the extract on a given cell (bacterial, animal tissues, or human tissues), the test should be repeated, because each cell acquires a different specific immunity under the given environmental conditions.

## 5. Conclusions

The conducted research allowed for a clear conclusion to be defined about the presence of glucofrangulin A and B compounds in plant extracts. The concentrations of plant extracts, as well as their complexity and diversity, could be determined using the techniques of UV-VIS, FT-IR, and gas chromatography analysis. Microbiological studies showed the variability of the acquired resistance of the given microbiological strains to the given types of plant extracts containing anthranoid compounds.

The main conclusion from the analysis of the results is the positive effect of extracts on probiotic bacteria of the Lactobacillus genus. This allows for the assumption that the use of *F. alnus* Mill extracts will not have a direct negative effect on the microflora. At the same time, it is interesting to observe that bacteria of the genus *P. fluorescens* ATCC 17400 were sensitive to the extract. Moreover, studies on the influence of the extracts on the permeability of the cell membrane were carried out, indicating that contact with the extracts may reduce the total permeability of the membranes. This opens the possibility of using *F. alnus* Mill extracts as a factor regulating transport into cells, which may be used to support the activities of other bioactive substances.

## Figures and Tables

**Table 1:** Extraction efficiency parameters of *F. alnus* Mill. extracts.

Extractant	Polarity of the Extractant	m [g]	E [%]	λ_max1_ [nm]	A_max1_ [−]	λ_max2_ [nm]	A_max2_ [−]
Ethanol	0.654	0.99	19.78	221.5	0.34	265	0.20
Isopropanol	0.617	0.48	9.61	216	0.31	263	0.12
Methanol	0.762	0.96	19.23	217.5	0.31	265	0.18
Distilled water	1.000	0.61	12.22	219.5	0.33	265	0.19

m—extract dry mass; E—extraction efficiency; C—frangulin concentration in dry extract; λ_max1_, λ_max2_—wavelengths of peaks’ maxima; A_max1_, A_max2_—maximum absorption for the wave λ_max1_, λ_max2_.

## Data Availability

The raw/processed data required to reproduce these findings cannot be shared at this time, as the data also forms part of an ongoing study.

## Supplementary Materials

The following supporting information can be downloaded at: https://www.mdpi.com/article/10.3390/plants11202719/s1, Table S1. GC-TOFMS analysis - tetracyclic compounds of cholestane and cholate; BSTFA, N,O-Bis(trimethylsilyl)trifluoroacetamide, Ac2O—acetic anhydride; MeOH—methanol, EtOH—ethanol, i-PrOH—isopropanol; Table S2. GC-TOFMS analysis—examples of chemical compounds present in plant solutions; BSTFA, N,O-Bis(trimethylsilyl)trifluoroacetamide, Ac2O—acetic anhydride; MeOH—methanol, EtOH—ethanol, i-PrOH—isopropanol; Table S3. GC-TOFMS analysis—examples of other compounds present in plant extracts after derivatization with BSTFA. References [36,37,38,39,40,41,42,43,44,45] are cited in the supplementary materials.
