# Supplementary material for: Extracts from Frangula alnus Mill. and Their Effects on Environmental and Probiotic Bacteria
Source: Plants (Basel). 2022 Oct 14;11(20):2719. doi: 10.3390/plants11202719 (PMC9607076; doi:10.3390/plants11202719)
Supplement: Supplementary file 1 [file plants-11-02719-s001.zip › plants-1876052-supplementary.pdf]

plants

Article

Effects on Environmental and Probiotic Bacteria of Extracts from the Natural Laxative – Bark of *Frangula alnus* Mill.

Agata Kledecka [1], Przemysław Siejak [2], Anubhav Pratap-Singh [3], Przemysław Łukasz Kowalczewski [4], Farahnaz Fathordoobady[3], Maciej Jarzębski [2], and Wojciech Smułek [1,*]

[1] Institute of Chemical Technology and Engineering, Poznan University of Technology, 4 Berdychowo St., 60-965 Poznań, Poland
[2] Department of Physics and Biophysics, Poznań University of Life Sciences, 38/42 Wojska Polskiego St., 60-637 Poznań, Poland
[3] Food Nutrition and Health Program, The University of British Columbia, 2205 East Mall, Vancouver, BC V6T 1Z4, Canada
[4] Department of Food Technology of Plant Origin, Poznań University of Life Sciences, 31 Wojska Polskiego St., 60-637 Poznań, Poland
* Correspondence: wojciech.smulek@put.poznan.pl

1. Gas Chromatography – Mass Spectrometry

The next stage of the research was devoted to the identification of bioactive compounds present in the extracts in addition to glucofrangulin. The most important of the identified compounds are summarized in Tables S1-S3 below.

Table S1. GC-TOFMS analysis - tetracyclic compounds of cholestane and cholate; BSTFA, N,O-Bis(trimethylsilyl)trifluoroacetamide, Ac2O – acetic anhydride; MeOH – methanol, EtOH – ethanol, i-PrOH – isopropanol.

Derivatization reagent	Extractant	Compound identified
BSTFA	H2O	Cholest-4-ene-3,6-dione
BSTFA	H2O	Cholest-5-en-3-ol, 6-nitro-, acetate (ester), á)-
Ac2O	MeOH, i-PrOH<br>H2O	
BSTFA	MeOH	Cholestan-3-ol, 5-chloro-6-nitro-, acetate (ester), (3á,5à,6á)-
BSFTA	i-PrOH	Cholan-24-oic acid, 3,7-dioxo-, (5β)-
BSTFA	EtOH	Cholestan-3-ol, 5-chloro-6-nitro-, (3β,5α,6β)-
Ac2O	i-PrOH	
Ac2O	MeOH, H2O	Cholestan-3-ol, 5-chloro-6-nitro-, acetate (ester), (3β,5α,6β)-
Ac2O	i-PrOH	5-Chloro-6beta-nitro-5alpha-cholestan-3-one
Ac2O	H2O	Cholan-24-oic acid, 3,7-dioxo-, (5β)-

Table S2. GC-TOFMS analysis - examples of chemical compounds present in plant solutions; BSTFA, N,O-Bis(trimethylsilyl)trifluoroacetamide, Ac2O – acetic anhydride; MeOH – methanol, EtOH – ethanol, i-PrOH – isopropanol.

Derivatization Reagent	Extractant	Compound Identified	Known biological activity
BSTFA	EtOH		-

Derivatization Reagent	Extractant	Compound Identified	Known biological activity
BSTFA Ac_2O	H_2O	8a-(Acetyloxy)-2a-[(acetyloxy)methyl]-6b-hydroxy-3a-methoxy-1,1,5,7-tetramethyl-4-oxo-1,1a,1b,1c,2a,3,3a,4,6a,6b,7,8-dodecahydro-8ah-cyclopropa[5',6']benzo[1',2':7,8]azuleno[5,6-b]oxiren-8-yl acetate	
BSTFA	H_2O, MeOH, EtOH	1,3,5-Triazine, 2-(hexylthio)-4,6 bis(trichloromethyl)-	-
Ac_2O	H_2O		
BSTFA	H_2O	Ethyl iso-allocholate	Fungicidal activity compound [1]
BSTFA	MeOH		
BSTFA	MeOH	(5á)Pregnane-3,20á-diol, 14à,18à-[4-methyl-3-oxo-(1-oxa-4-azabutane-1,4-diyl)]-	
Ac_2O	EtOH, MeOH, H_2O	(5á)Pregnane-3,20á-diol, 14à,18à-[4-methyl-3-oxo-(1-oxa-4-azabutane-1,4-diyl)]-, diacetate	-
BSTFA	H_2O	3-Pyridinecarboxylic acid derivative	-
Ac_2O	EtOH, i-PrOH, MeOH, H_2O		
Ac_2O	EtOH		
BSTFA	H_2O	Arecoline	It is a pyridine alkaloid with a psychoactive effect [2]
Ac_2O	MeOH		
Ac_2O	MeOH, H_2O	à-D-Glucopyranoside, methyl 2-(acetylamino)-2-deoxy-3-O-(trimethylsilyl)-, cyclic methylboronate	-

Table S3. GC-TOFMS analysis - examples of other compounds present in plant extracts after derivatization with BSTFA.

Extractant	Compound Identified	Known biological activity
EtOH	Apigenin 8-C-glucoside (vitexin)	It inhibits thyroid peroxidase [3]
	Etidronic acid	It stabilizes easily oxidizable compounds, it inhibits resorption of bone via a number of cellular mechanisms [6]
	(-) Quinic acid	It is an astringent and is also a starting material for the synthesis of pharmaceuticals [7]
MeOH	Ala-Gly	Dipeptide composed of two amino acid residues alanine and glycine [8]
	4-Hydroxyanthraquinone-2-carboxylic acid, di-TMS	Derivative of an anthraquinone compound [9]

References

1. Abubacker, M.N.; Devi, P.K. In Vitro Antifungal Potentials of Bioactive Compound Oleic Acid, 3-(Octadecyloxy) Propyl Ester Isolated from Lepidagathis Cristata Willd. (Acanthaceae) Inflorescence. *Asian Pacific Journal of Tropical Medicine* **2014**, *7*, S190–S193, doi:10.1016/S1995-7645(14)60230-3.
2. Papke, R.L.; Horenstein, N.A.; Stokes, C. Nicotinic Activity of Arecoline, the Psychoactive Element of "Betel Nuts", Suggests a Basis for Habitual Use and Anti-Inflammatory Activity. *PLoS ONE* **2015**, *10*, e0140907, doi:10.1371/journal.pone.0140907.
3. Gaitan, E.; Cooksey, R.C.; Legan, J.; Lindsay, R.H. Antithyroid Effects in Vivo and in Vitro of Vitexin: A C-Glucosylflavone in Millet. *The Journal of Clinical Endocrinology & Metabolism* **1995**, *80*, 1144–1147, doi:10.1210/jcem.80.4.7714083.
4. Numazawa, S.; Honma, Y.; Yamamoto, T.; Yoshida, T.; Kuroiwa, Y. A Cardiotonic Steroid Bufalin-like Factor in Human Plasma Induces Leukemia Cell Differentiation. *Leukemia Research* **1995**, *19*, 945–953, doi:10.1016/0145-2126(95)00081-X.
5. Dalbeth, N.; Lauterio, T.J.; Wolfe, H.R. Mechanism of Action of Colchicine in the Treatment of Gout. *Clinical Therapeutics* **2014**, *36*, 1465–1479, doi:10.1016/j.clinthera.2014.07.017.
6. Ariyoshi, T.; Eishi, K.; Sakamoto, I.; Matsukuma, S.; Odate, T. Effect of Etidronic Acid on Arterial Calcification in Dialysis Patients: *Clinical Drug Investigation* **2006**, *26*, 215–222, doi:10.2165/00044011-200626040-00006.
7. Wen, M.; Han, Z.; Cui, Y.; Ho, C.-T.; Wan, X.; Zhang, L. Identification of 4-O-p-Coumaroylquinic Acid as Astringent Compound of Keemun Black Tea by Efficient Integrated Approaches of Mass Spectrometry, Turbidity Analysis and Sensory Evaluation. *Food Chemistry* **2022**, *368*, 130803, doi:10.1016/j.foodchem.2021.130803.
8. Yu, C.P.; Gerlei, K.Z.; Rágyanszki, A.; Jensen, S.J.K.; Viskolcz, B.; Csizmadia, I.G. Reactivity of Ala-Gly Dipeptide with β-Turn Secondary Structure. *Chemical Physics Letters* **2018**, *692*, 402–406, doi:10.1016/j.cplett.2017.12.057.
9. Czyżewska, U.; Konończuk, J.; Teul, J.; Drągowski, P.; Pawlak-Morka, R.; Surażyński, A.; Miltyk, W. Verification of Chemical Composition of Commercially Available Propolis Extracts by Gas Chromatography–Mass Spectrometry Analysis. *Journal of Medicinal Food* **2015**, *18*, 584–591, doi:10.1089/jmf.2014.0069.
10. Yu, S.P.; Choi, D.W. Na^{+}-Ca^{2+} Exchange Currents in Cortical Neurons: Concomitant Forward and Reverse Operation and Effect of Glutamate. *European Journal of Neuroscience* **1997**, *9*, 1273–1281, doi:10.1111/j.1460-9568.1997.tb01482.x.
